# A Novel Dietary Index for Gut Microbiota (DI‐GM) is Associated With Inflammation, Mental Health, and Tumor Biomarkers in Adults: A Cross‐Sectional Study

**DOI:** 10.1002/fsn3.70951

**Published:** 2025-10-24

**Authors:** Yaqin Meng, Weiyu Ma, Xiuxiu Li, Ninggang Zhang

**Affiliations:** ^1^ Department of Psychiatry The First Hospital of Shanxi Medical University Taiyuan Shanxi China; ^2^ Department of Gastroenterology, Shanxi Province Cancer Hospital/Shanxi Hospital Affiliated to Cancer Hospital, Chinese e Academy of Medical Sciences/Cancer Hospital Affiliated to Shanxi Medical University Taiyuan Shanxi China

**Keywords:** depression, dietary index, gut microbiota, inflammation, tumor biomarkers

## Abstract

The interplay between diet, gut microbiota, and mental health has garnered increasing attention. This study aimed to examine the association of a novel Dietary Index for Gut Microbiota (DI‐GM) with depression, anxiety, and intestinal tumor biomarkers, and to explore whether gut microbiota composition mediates these associations. A cross‐sectional study was conducted among 650 Chinese adults aged 18–65 years. DI‐GM scores were calculated based on habitual dietary intake. Mental health was assessed using PHQ‐9, STAI‐A, PSQI, and WHO‐5. Gut microbiota metrics (Firmicutes/Bacteroidetes [F/B] ratio, alpha diversity), inflammatory markers (CRP, calprotectin, TMAO), brain‐derived neurotrophic factor (BDNF), and carcinoembryonic antigen (CEA) were measured. Linear regression and structural equation modeling were used to assess associations and mediation effects. Higher DI‐GM scores were significantly associated with lower BMI, improved lipid profiles (↑ HDL‐C: *β* = 0.41; 95% CI: 0.39–0.44), reduced insulin resistance (↓ HOMA‐IR: *β* = −2.11; 95% CI: −2.15 to −2.07), and increased BDNF (*β* = 0.25; 95% CI: 0.19–0.30). Participants in the higher quartiles had lower depressive (PHQ‐9: *β* = −0.32; 95% CI: −0.46 to −0.17) and anxiety scores (STAI‐A: *β* = −0.08; 95% CI: −0.10 to −0.06). Inflammatory markers including CRP, TMAO, and CEA were significantly reduced. The F/B ratio was inversely associated with DI‐GM (*p* < 0.001) and partially mediated its relationship with depression (*β* = −0.013) and anxiety (*β* = −0.07). The DI‐GM score is a promising dietary tool linked to favorable metabolic, inflammatory, and psychological outcomes, partially mediated by gut microbiota.

## Graphical abstract

The graphical abstract illustrates the impact of the DI‐GM score on various aspects of human health. A high DI‐GM score is associated with increased microbial diversity and decreased F/B (Firmicutes to Bacteroidetes) ratio in the gut microbiome. This in turn leads to beneficial effects, including reduced LDL cholesterol and insulin resistance (HOMA‐IR), increased brain‐derived neurotrophic factor (BDNF) levels, and decreased depression and anxiety, as well as decreased markers of inflammation (CRP, and calprotectin) and cancer (CEA). The results suggest that the changes in the gut microbiota's F/B ratio partially mediate the observed psychological and inflammatory effects.

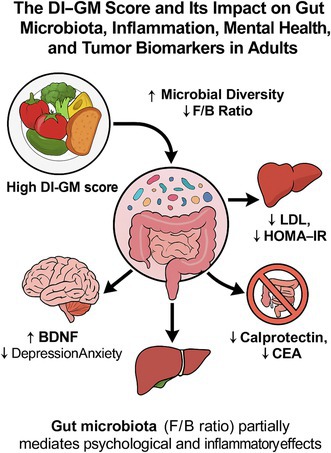

## 1 Introduction

The gut microbiota has emerged as a pivotal regulator of both physical and mental health, influencing diverse physiological systems through the so‐called “gut‐brain axis” (Rutsch et al. 2020). Accumulating evidence underscores the role of gut microbial communities in modulating immune function, metabolic homeostasis, neurotransmitter production, and neuroinflammatory responses—processes increasingly implicated in the pathophysiology of psychiatric disorders and gastrointestinal malignancies (Estrada and Contreras 2019; Maranduba et al. 2015; Ağagündüz et al. 2023). Diet, as one of the most influential environmental determinants of gut microbiota composition and function, offers a promising avenue for modulating these pathways through lifestyle interventions (Bianchetti et al. 2023; Kolodziejczyk et al. 2019).

Dietary patterns rich in fiber, polyphenols, and fermented foods have been associated with increased microbial diversity and the enrichment of beneficial taxa such as *Bifidobacterium* and *Lactobacillus*, which are known to produce short‐chain fatty acids (SCFAs) and exert anti‐inflammatory effects (Annunziata et al. 2020; Fusco et al. 2023). Conversely, diets high in refined sugars, saturated fats, and processed meats have been linked to microbial dysbiosis, characterized by a reduced abundance of symbiotic bacteria and an increased prevalence of pro‐inflammatory species (Bengmark 2013; Jian et al. 2021). This imbalance may contribute to systemic inflammation, impaired intestinal barrier integrity, and altered signaling to the central nervous system—mechanisms that have been implicated in both mood disorders and colorectal tumorigenesis (Zeng et al. 2017; Buttó and Haller 2016; Mostafavi Abdolmaleky and Zhou 2024).

Psychological distress, particularly depression and anxiety, is highly prevalent worldwide and is increasingly recognized as having a bidirectional relationship with gut health (Mofrad et al. 2019; Effatpanah et al. 2024). Notably, alterations in gut microbiota composition and function have been observed in individuals with major depressive disorder, suggesting that microbial dysbiosis may be both a contributor to and a consequence of psychological stress (Cheng et al. 2025). Similarly, chronic low‐grade inflammation and microbial metabolites such as trimethylamine N‐oxide (TMAO) have been implicated in the development of neuropsychiatric symptoms (Xie et al. 2025) and early stages of colorectal cancer (Zhou et al. 2024).

Despite growing interest in diet–microbiota interactions, there remains a need for comprehensive, population‐applicable dietary indices that integrate both favorable and unfavorable dietary components relevant to gut microbial ecology. In this context, we applied the Dietary Index for Gut Microbiota (DI‐GM)—a previously developed composite score reflecting adherence to a microbiota‐supportive dietary pattern based on current scientific evidence regarding diet‐induced microbial changes (Kase et al. 2024). Although one previous study assessed the association between the DI‐GM and depressive symptoms [20], no study to date has examined its relationship with gut microbiota composition, diversity, or intestinal tumor biomarkers. This gap highlights the need to understand how a microbiota‐targeted dietary index may influence microbial ecology and early biomarkers, thereby informing integrative prevention strategies for mental and gastrointestinal disorders.

Moreover, no prior research has explored the potential mediating role of gut microbiota profiles, inflammatory biomarkers (e.g., CRP, fecal calprotectin), and microbial metabolites (e.g., TMAO, BDNF) in linking diet to psychological and tumor‐related outcomes. By identifying these mechanisms, we can move beyond simple associations and begin to elucidate potential biological pathways that may underlie diet‐related risk or resilience.

This study investigates the association between the newly developed DI‐GM and mental health indicators—namely depression, anxiety, and well‐being—as well as early biomarkers of intestinal tumorigenesis. It also explores whether gut microbiota composition, systemic inflammation, and microbial metabolites mediate these relationships. To our knowledge, this is the first comprehensive study linking a gut microbiota‐specific dietary index to both psychological outcomes and tumor‐related biomarkers in a large adult population.

## 2 Methods

### 2.1 Study Design and Participants

This cross‐sectional study was conducted on a sample of 650 Chinese adults aged 18 to 65 years, recruited from various health centers and community settings to ensure representation across diverse socioeconomic and lifestyle backgrounds. Efforts were made to minimize bias and ensure methodological rigor throughout data collection. Dietary assessments were standardized using trained personnel, validated tools, and visual aids to reduce recall bias. Stool sample collection, DNA extraction, and sequencing procedures followed established protocols to ensure reproducibility and reliability. Psychological assessments were administered by trained professionals using psychometrically validated instruments.

**FIGURE 1:**
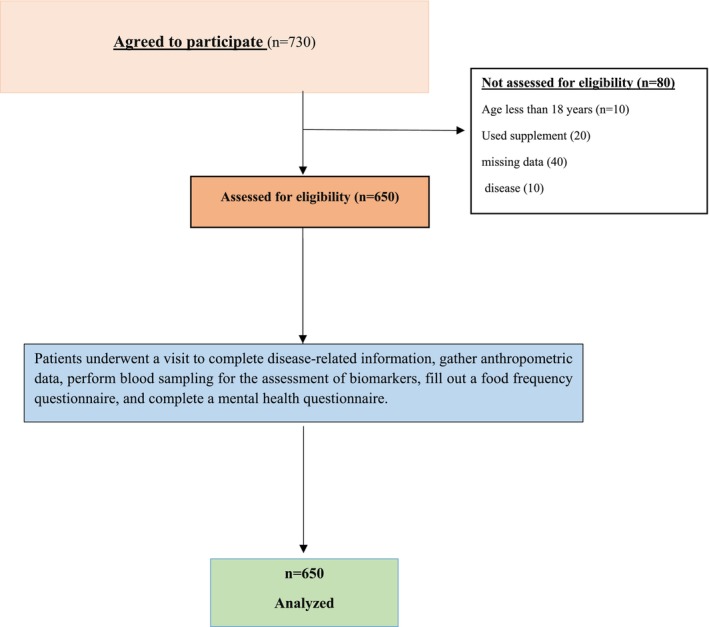
Flowchart of study participants' enrollment and inclusion.

Participants were recruited using stratified convenience sampling. Inclusion criteria included: Chinese nationality, age between 18 and 65 years, nonpregnant and nonlactating status, not postmenopausal, and no history of diagnosed depression, psychiatric disorders, or chronic diseases. Eligibility was confirmed through structured interviews and clinical screening conducted by trained staff. Participants were also required to be free from recent (< 3 months) antibiotic or probiotic use, which could confound gut microbiota composition. Individuals were excluded if they had a history of significant life events within the past year, reported energy intakes outside 800–4200 kcal/day (2092–14,644 kJ/day), or had used antibiotics or probiotic supplements in the past three months. Participants adhering to restrictive or specialized dietary regimens (e.g., vegan, vegetarian, ketogenic) were excluded to minimize dietary confounding effects on gut microbiota composition and reduce dietary heterogeneity. Written informed consent was obtained from all participants. Of the 730 individuals initially screened, 80 were excluded due to missing data or exclusion criteria. Final analysis included 650 participants (Flowchart, Figure 1).

The study protocol was approved by the Medical Research Ethics Committee of Shanxi Province Cancer Hospital (NO; 2024–417), in accordance with the Declaration of Helsinki. Participant recruitment, consent, and data collection procedures were standardized across all centers to minimize site‐related bias.

### 2.2 Sample Size Calculation

The required sample size was calculated assuming a prevalence (P) of 29% (Mofrad et al. 2019), a margin of error (d) of 4.03%, and a significance level of α = 0.05. The minimum sample size was estimated at 590 participants. An additional 10% was added to account for possible dropouts or incomplete data, yielding a final target of 650 participants.

### 2.3 Dietary Assessment and DI‐GM Construction

Dietary intake was assessed using three nonconsecutive 24‐h dietary recalls (including one weekend day), administered by trained nutritionists employing visual aids and portion size estimation tools. To enhance recall accuracy, interviews were scheduled within 24 h of food consumption and cross‐checked with food diaries when available. Dietary data were analyzed using a validated, region‐specific food composition database.

The DI‐GM was constructed based on existing literature. The DI‐GM incorporated both favorable (e.g., total dietary fiber, fermented foods, polyphenol‐rich fruits and vegetables) and unfavorable (e.g., added sugars, saturated fats, processed meats) dietary components known to influence gut microbiota diversity and function. Scores were calculated by summing standardized intakes of these components, with higher DI‐GM scores reflecting greater adherence to a gut‐supportive dietary pattern. Component weights were derived from prior cohort studies and expert recommendations on dietary factors influencing gut microbiome health (Kase et al. 2024).

To minimize information bias, data entry was double‐checked, and nutritionists were blinded to participants' psychological and biomarker data.

### 2.4 Psychological Assessment

The psychological status of participants was comprehensively evaluated using three validated, standardized self‐report instruments that assess depression, anxiety, and subjective well‐being. These tools were selected based on their extensive use in epidemiological research and their demonstrated reliability and validity in diverse populations, including Chinese cohorts (Zhou et al. 2024; Du et al. 2022; Wang et al. 2022).

Depression levels were measured using the Patient Health Questionnaire‐9 (PHQ‐9), a recognized screening tool based on DSM‐IV diagnostic criteria. The PHQ‐9 includes nine items that assess the frequency of depressive symptoms over the preceding two weeks, rated on a 4‐point Likert scale ranging from 0 (“not at all”) to 3 (“nearly every day”). Total scores range from 0 to 27, with thresholds of 5, 10, 15, and 20 indicating mild, moderate, moderately severe, and severe depression, respectively (Manea et al. 2015).

Anxiety symptoms were measured using the State–Trait Anxiety Inventory (STAI), a widely used self‐report instrument designed to assess both temporary (state) and more enduring (trait) anxiety. The STAI consists of two 20‐item subscales, each scored on a 4‐point Likert scale, with higher scores indicating greater anxiety (Spielberger and Rickman 1988).

Sleep quality was examined using the Pittsburgh Sleep Quality Index (PSQI), a validated self‐administered tool that captures various aspects of sleep, including subjective latency, quality, duration, disturbances, efficiency, use of sleep medication, and daytime dysfunction over the past month (Mollayeva et al. 2016).

Psychological well‐being was assessed using the World Health Organization‐Five Well‐Being Index (WHO‐5), a brief, positively worded instrument consisting of five items reflecting mood, energy, and overall life satisfaction. Participants rated how often they experienced each feeling during the past two weeks on a 6‐point scale (0 = “at no time” to 5 = “all of the time”) (Dadfar et al. 2018).

All psychological instruments used were previously validated in Chinese e populations, demonstrating strong psychometric properties (Cronbach's alpha > 0.80). Trained research psychologists, blinded to participants' dietary and microbiota data, conducted assessments in quiet, private settings to minimize bias. Participants were assured of confidentiality and encouraged to respond honestly. Questionnaires were reviewed in real time to address missing or unclear responses; for cases with < 5% missing data, mean imputation was used if at least 80% of the scale was completed.

### 2.5 Microbiota Profile

Participants provided fresh stool samples using sterile kits, which were transported under cold chain conditions and stored at −80°C. DNA was extracted using the QIAamp DNA Stool Mini Kit (Qiagen, Germany), with slight protocol optimizations to enhance yield. Microbiota composition was analyzed via 16S rRNA gene sequencing targeting the V3–V4 regions on the Illumina MiSeq platform. Data processing and taxonomic assignment were performed using QIIME2. Reads were quality filtered, de‐multiplexed, and assigned to operational taxonomic units (OTUs) at 97% similarity. Key outcomes included alpha diversity (Shannon, Chao1) and the F/B ratio to Bacteroidetes (F/B) ratio. Negative controls, technical replicates, and mock community standards were included to monitor batch effects and sequencing accuracy (Costa and Weese 2019).

### 2.6 Biomarker Analysis

Biological samples were collected to assess markers of systemic inflammation, neuroendocrine function, microbial metabolism, and tumorigenesis. Serum levels of CRP and BDNF were measured using ELISA kits (R&D Systems, Minneapolis, MN). Plasma TMAO concentrations were determined via high‐performance liquid chromatography (HPLC). Fecal calprotectin and serum carcinoembryonic antigen (CEA) were analyzed using commercial immunoassays. All assays reported intra‐ and inter‐assay variability of < 10%. To minimize measurement bias, laboratory technicians were blinded to participants' dietary and psychological profiles.

### 2.7 Statistical Analysis

All statistical analyzes were conducted using Stata 16.0 (StataCorp, College Station, TX, USA), and two‐sided *p*‐values < 0.05 were considered statistically significant. Descriptive statistics were used to summarize baseline demographic, dietary, psychological, microbial, and biomarker data. The normality of continuous variables was assessed using the Shapiro–Wilk test. Normally distributed variables were presented as means ± standard deviations (SD), while nonnormally distributed variables were reported as medians with interquartile ranges (IQRs). Categorical variables were expressed as frequencies and percentages. Associations between DI‐GM scores and psychological outcomes—including depressive symptoms (PHQ‐9) and anxiety levels (STAI)—were examined using multiple linear regression models, adjusted for potential confounders including age, sex, body mass index (BMI), total energy intake, and physical activity (measured in METs). Effect estimates were reported as unstandardized beta coefficients with corresponding 95% confidence intervals (CIs).

Mediation analyzes were performed using structural equation modeling (SEM) to assess whether the F/B ratio mediated the association between DI‐GM scores and key outcomes (PHQ‐9, STAI, CRP, BDNF, and PSQI). The indirect effect was estimated by multiplying the coefficient of the path from DI‐GM to the F/B ratio by the coefficient of the path from the F/B ratio to the outcome variable. Structural equation modeling (SEM) with maximum likelihood estimation was performed to assess direct and indirect effects of DI‐GM score on outcomes via the F/B ratio, with model fit evaluated using RMSEA, CFI, TLI, and SRMR indices following established guidelines.

## 3 Results

### 3.1 Participant Characteristics by DI‐GM Score Quartiles

A total of 650 participants were stratified into quartiles based on their DI‐GM scores, reflecting adherence to a dietary pattern associated with gut‐immune health. As expected, there was a significant increase in DI‐GM score across quartiles (Q1: 5.28 ± 0.7; Q3: 11.36 ± 0.48; *p* < 0.001), confirming effective stratification. No significant differences were observed in age or physical activity levels across groups. Socioeconomic status (SES) showed marginal variation across quartiles (*p* = 0.051), with higher SES individuals more frequently represented in Q3. Dietary intake patterns revealed a strong inverse relationship between DI‐GM score and consumption of calories, carbohydrates, protein, fat, and fiber intakes. All comparisons were statistically significant (*p* < 0.001), as detailed in Table 1. These findings indicate that a higher DI‐GM score is reflective of a nutrient‐dense, fiber‐rich, and lower‐energy diet. Regarding micronutrients and fatty acids, magnesium, PUFA, MUFA, and omega‐3 fatty acids increased significantly across quartiles, with magnesium rising from 268 mg/day in Q1 to 388 mg/day in Q3, and omega‐3 from 1.45 to 1.93 g/day. Saturated fatty acids decreased from 16.9 g/day in Q1 to 13.1 g/day in Q3. These results suggest that higher DI‐GM scores are associated with healthier dietary patterns characterized by higher fiber, unsaturated fats, and essential minerals.

**TABLE 1:** Demographics, lifestyle, and dietary factors of study participants by DI‐GM score quartiles (*N* = 650).

Variables	Total (*N* = 650)	Q1 (*N* = 204)	Q2 (*N* = 281)	Q3 (*N* = 165)	*p*
DI‐GM score (mean)	8.47 ± 2.48	5.28 ± 0.70	9.10 ± 1.06	11.36 ± 0.48	< 0.001
Age (years)	37.64 ± 10.10	37.56 ± 6.60	37.00 ± 10.37	38.83 ± 12.83	0.547
BMI	23.77 ± 1.21	23.67 ± 1.4	23.82 ± 1.25	23.78 ± 0.83	0.408
Physical activity (total MET)	12.46 ± 1.64	12.56 ± 1.96	12.48 ± 1.25	12.30 ± 1.79	0.129
Total energy (kcal/day)	2192.62 ± 181.18	2372.06 ± 128.09	2144.48 ± 150.40	2052.73 ± 85.22	< 0.001
Carbohydrate (g/day)	300.80 ± 19.78	318.73 ± 19.89	297.94 ± 12.47	283.52 ± 8.90	< 0.001
Protein (g/day)	94.65 ± 13.60	108.63 ± 9.56	92.79 ± 8.49	80.55 ± 6.85	< 0.001
Fat (g/day)	66.99 ± 13.85	79.29 ± 4.17	67.05 ± 11.51	51.70 ± 9.40	< 0.001
Fiber (g/day)	27.90 ± 7.24	21.42 ± 4.23	27.43 ± 5.37	36.72 ± 2.28	< 0.001
Iron (mg/day)	12.61 ± 2.24	12.79 ± 1.80	12.84 ± 2.01	12.00 ± 2.89	< 0.001
Magnesium (mg/day)	324.64 ± 87.45	267.51 ± 82.21	329.12 ± 64.44	387.63 ± 81.93	< 0.001
PUFA (g/day)	17.1 ± 2.34	14.57 ± 0.55	17.63 ± 2.05	19.33 ± 0.86	< 0.001
MUFA (g/day)	14.11 ± 2.37	11.62 ± 0.91	14.63 ± 2.05	16.29 ± 1.06	< 0.001
Omega‐3 fatty acids (g/day)	1.71 ± 0.23	1.45 ± 0.065	1.76 ± 0.20	1.93 ± 0.08	< 0.001
Saturated Fatty Acids (g/day)	15.56 ± 2.65	16.90 ± 0.4	16.05 ± 0.41	13.07 ± 4.31	< 0.001

*Note:* Values are presented as mean ± SD or *n* (%). *p*‐values were calculated using ANOVA for continuous variables and chi‐square tests for categorical variables. Variables with *p* < 0.05 indicate statistically significant differences across DI‐GM score quartiles. Abbreviations: MET, metabolic equivalent task; SES, socioeconomic status.

**TABLE 2:** Biochemical and physiological measurements by DI‐GM score quartiles.

Variables	Total (*N* = 650)	Q1 (*N* = 204)	Q2 (*N* = 281)	Q3 (*N* = 165)	*p*
TG (mg/dL)	129.97 ± 4.93	135.17 ± 3.24	128.40 ± 3.29	126.21 ± 3.58	< 0.001
LDL (mg/dL)	95.18 ± 6.74	100.71 ± 4.46	93.53 ± 6.93	91.15 ± 3.78	< 0.001
TC (mg/dL)	186.64 ± 11.81	197.44 ± 7.34	184.74 ± 11.13	176.50 ± 4.32	< 0.001
HDL (mg/dL)	51.32 ± 7.04	43.72 ± 1.62	52.92 ± 6.15	58.01 ± 2.59	< 0.001
Diastolic blood pressure (mmHg)	81.87 ± 4.15	85.74 ± 3.58	81.29 ± 2.62	78.09 ± 2.64	< 0.001
Systolic blood pressure (mmHg)	128.19 ± 8.30	137.16 ± 5.25	125.65 ± 5.84	121.42 ± 4.8	< 0.001
Fasting Glucose (mg/dL)	91.24 ± 4.2	95.17 ± 3.61	90.81 ± 2.52	87.10 ± 2.47	< 0.001
Fasting Insulin (μU/mL)	6.08 ± 1.76	7.87 ± 1.46	5.63 ± 1.33	4.64 ± 0.48	< 0.001
HOMA‐IR	1.42 ± 0.37	1.80 ± 0.26	1.34 ± 0.31	1.10 ± 0.10	< 0.001
BDNF (ng/mL)	11.22 ± 2.25	9.42 ± 1.84	11.56 ± 1.64	12.89 ± 2.03	< 0.001
PHQ‐9	7.589 ± 1.16	8.57 ± 0.50	7.45 ± 1.22	6.58 ± 0.49	< 0.001
STAI	4.6 ± 0.96	5.57 ± 0.72	4.25 ± 0.73	4.00 ± 0.59	< 0.001
Sleep Quality (PSQI)	3.58 ± 0.83	3.44 ± 0.76	3.68 ± 0.87	3.57 ± 0.85	0.055

*Note:* Values are presented as mean ± SD. *p*‐values reflect comparisons across DI‐GM score quartiles using ANOVA. Significant differences across groups are indicated by *p* < 0.05. Abbreviations: BDNF, brain‐derived neurotrophic factor; HDL, high‐density lipoprotein; HOMA‐IR, homeostatic model assessment of insulin resistance; LDL, low‐density lipoprotein; PHQ‐9, patient health questionnaire‐9; PSQI, Pittsburgh Sleep Quality Index; STAI, state–trait anxiety inventory; TC, total cholesterol; TG, triglycerides.

### 3.2 Biochemical and Physiological Profiles Across DI‐GM Score Groups

Table 2 outlines the cardiometabolic, metabolic, and mental health profiles across DI‐GM score quartiles. Participants in Q1 exhibited significantly elevated levels of TG, LDL‐C, TC, fasting glucose, insulin, and HOMA‐IR compared to those in Q3, indicating an adverse metabolic profile among individuals with lower DI‐GM scores. Conversely, HDL‐C increased with DI‐GM score, suggesting improved lipid metabolism. BDNF levels increased with DI‐GM score, potentially reflecting enhanced neurocognitive function. All reported differences were statistically significant (*p* < 0.001).

Mental health outcomes, assessed via PHQ‐9 and STAI, were significantly better in higher DI‐GM score groups. PHQ‐9 scores decreased from a mean of 8.57 in Q1 to 6.58 in Q3 (*p* < 0.001), while STAI scores declined from 5.57 in Q1 to 4.00 in Q3 (*p* < 0.001), indicating improvements in both depressive and anxiety symptoms across quartiles. Sleep quality, measured by the PSQI, showed a slight improvement from Q1 to Q3; however, the difference did not reach statistical significance (*p* = 0.055).

### 3.3 Gut Microbiota Composition and Inflammatory Markers by DI‐GM Score

Table 3 presents associations between DI‐GM score and markers of gut microbiota composition and inflammation. Higher DI‐GM scores were consistently linked with reduced systemic and intestinal inflammation, as evidenced by lower levels of CRP, TMAO, LPS, CEA, and calprotectin lower across quartiles. These associations were all statistically significant (*p* < 0.001). The F/B ratio, a commonly used proxy for microbial dysbiosis, was significantly lower in Q3 compared to Q1 (*p* < 0.001), indicating a shift toward a more balanced microbial community with increasing DI‐GM score. Alpha diversity, as measured by the Shannon index, showed a statistically significant decrease with higher DI‐GM scores (*p* < 0.001), although this may reflect changes in specific taxa rather than overall diversity loss and warrants further exploration.

**TABLE 3:** Gut microbiota, inflammation, and tumor‐associated markers by DI‐GM score quartiles.

Variables	Total (*N* = 650)	Q1 (*N* = 204)	Q2 (*N* = 281)	Q3 (*N* = 165)	*p*
CRP (mg/L)	0.88 ± 0.32	1.26 ± 0.18	0.77 ± 0.23	0.61 ± 0.12	< 0.001
TMAO (μM)	1.58 ± 0.43	2.13 ± 0.19	1.38 ± 0.25	1.24 ± 0.19	< 0.001
LPS	11.32 ± 2.13	13.29 ± 1.28	10.81 ± 1.99	9.76 ± 1.21	< 0.001
CEA (ng/mL)	1.99 ± 0.66	2.64 ± 0.33	1.78 ± 0.62	1.55 ± 0.38	< 0.001
Calprotectin (μg/mL)	51.33 ± 6.13	58.44 ± 3.58	49.46 ± 3.15	45.72 ± 3.97	< 0.001
F/B ratio	1.10 ± 1.18	1.39 ± 1.74	1.00 ± 0.99	0.89 ± 0.08	< 0.001
Shannon Index	3.19 ± 0.6	3.31 ± 0.58	3.12 ± 0.60	3.14 ± 0.61	0.001

*Note:* Values are presented as mean ± SD or *n* (%), as appropriate. *p*‐values were derived from ANOVA (continuous variables) or chi‐square tests (categorical variables). Statistically significant differences across quartiles are indicated by *p* < 0.05. Abbreviations: CEA, Carcinoembryonic antigen; CRP, C‐reactive protein; F/B ratio, Firmicutes‐to‐Bacteroidetes ratio; LPS, Lipopolysaccharide; TMAO, Trimethylamine N‐oxide.

### 3.4 Linear Regression Analysis of DI‐GM Score With Inflammatory and Cardiometabolic Biomarkers

Table 4 demonstrates significant associations between the Dietary Index for Gut Microbiota (DI‐GM) and key metabolic, inflammatory, and mental health biomarkers. Higher DI‐GM scores were linked to improved glycemic control, including lower fasting glucose (*β* = −0.49), insulin (*β* = −0.20), and HOMA‐IR (*β* = −2.11), with effects remaining significant after full adjustment for age, sex, BMI, calorie, and fiber intake. Inflammatory markers also decreased significantly—CRP (Model 2: *β* = −2.19), TMAO (*β* = −0.24), LPS (*β* = −8.51), and CEA (*β* = −2.94)—suggesting that a gut microbiota‐supportive diet reduces systemic and intestinal inflammation.

**TABLE 4:** Linear regression analysis of DI‐GM score with hepatic, inflammatory, and cardiometabolic biomarkers.

Dependent variables	Crude *β* (95% CI)	Model 1 *β* (95% CI)	Model 2 *β* (95% CI)	P for trend
Fasting glucose (mg/dL)	−0.49 (−0.52, −0.46)	−0.50 (−0.53, −0.46)	−0.49 (−0.52, −0.45)	< 0.001
Fasting insulin (μU/mL)	−0.20 (−0.21, −0.20)	−0.20 (−0.21, −0.20)	−0.20 (−0.21, −0.19)	< 0.001
HOMA‐IR	−2.11 (−2.15, −2.07)	−2.11 (−2.15, −2.07)	−2.10 (−2.14, −2.06)	< 0.001
CRP (mg/L)	−6.22 (−6.56, −5.87)	−4.58 (−5.20, −3.96)	−2.19 (−2.75, −1.63)	< 0.001
TMAO (μM)	−0.25 (−0.26, −0.24)	−0.25 (−0.26, −0.24)	−0.24 (−0.25, −0.23)	< 0.001
Total cholesterol (mg/dL)	−0.14 (−0.14, −0.14)	−0.14 (−0.14, −0.14)	−0.14 (−0.14, −0.13)	< 0.001
TG (mg/dL)	−0.07 (−0.07, −0.06)	−0.07 (−0.07, −0.06)	−0.06 (−0.06, −0.06)	< 0.001
LDL (mg/dL)	−0.14 (−0.15, −0.14)	−0.14 (−0.15, −0.14)	−0.14 (−0.15, −0.13)	< 0.001
TC (mg/dL)	−0.34 (−0.37, −0.32)	−0.14 (−0.14, −0.14)	−0.14 (−0.14, −0.13)	< 0.001
HDL (mg/dL)	0.43 (0.41, 0.46)	0.43 (0.41, 0.46)	0.41 (0.39, 0.44)	< 0.001
Diastolic blood pressure (mmHg)	−0.02 (−0.05, 0.02)	−0.02 (−0.05, 0.02)	−0.02 (−0.05, 0.01)	0.10
Systolic blood pressure (mmHg)	−0.00 (−0.02, 0.02)	−0.00 (−0.02, 0.02)	−0.00 (−0.02, 0.01)	0.60
LPS	−8.52 (−8.92, −8.12)	−8.51 (−8.88, −8.14)	−8.51 (−8.88, −8.14)	< 0.001
F/B ratio	−0.36 (−0.56, −0.16)	−0.09 (−0.22, 0.04)	−0.14 (−0.26, −0.02)	0.02
Shannon Index	0.12 (−0.58, 0.81)	0.11 (−2.59, 2.81)	−0.06 (−2.60, 2.58)	0.96
Simpson Score	−1.82 (−2.44, −1.20)	−1.72 (−1.24, −1.19)	−1.19 (−1.64, −1.74)	< 0.001
CEA (ng/mL)	−2.94 (−3.12, −2.76)	−1.98 (−2.32, −1.65)	−1.49 (−1.71, −1.27)	< 0.001
Calprotectin (μg/mL)	−0.35 (−0.36, −0.33)	−0.27 (−0.29, −0.24)	−0.17 (−0.18, −0.15)	< 0.001
BDNF (ng/mL)	0.79 (0.73, 0.85)	0.45 (0.38, 0.53)	0.25 (0.19, 0.30)	< 0.001
PHQ‐9	−1.57 (−1.68, −1.45)	−0.97 (−1.17, −0.78)	−0.32 (−0.46, −0.17)	< 0.001
State–trait anxiety inventory (STAI)	−0.21 (−0.23, −0.20)	−0.12 (−0.14, −0.10)	−0.08 (−0.10, −0.06)	< 0.001
Sleep quality (PSQI)	0.34 (0.11, 0.57)	0.07 (−0.07, 0.22)	0.04 (−0.06, 0.15)	0.40

*Note:* *β*‐coefficients represent the change in the dependent biomarker per 1‐unit increase in the DI‐GM score. Model 1: Adjusted for age, sex, MET (metabolic equivalent task), and BMI. Model 2: Model 1 + additional adjustment for total calorie and fiber intake. Crude: Unadjusted model. Confidence intervals (95% CI) that do not include zero indicate statistically significant associations at *p* < 0.05. Abbreviations: BDNF, brain‐derived neurotrophic factor; CEA, carcinoembryonic antigen; CR, C‐reactive protein; F/B, F/B ratio/Bacteroidetes ratio; HDL, high‐density lipoprotein; HOMA‐IR, homeostatic model assessment of insulin resistance; LDL, low‐density lipoprotein; LPS, lipopolysaccharide; PHQ‐9, Patient Health Questionnaire‐9; PSQI, Pittsburgh Sleep Quality Index; STAI, State–Trait Anxiety Inventory; TC, total cholesterol; TG, triglycerides; TMAO, trimethylamine N‐oxide.

Cardiometabolic outcomes showed favorable trends, with TC, LDL‐C, and TG decreasing and HDL‐C increasing with higher DI‐GM scores (Model 2: HDL‐C *β* = 0.41). Neurocognitive biomarkers improved as well, with BDNF levels rising (*β* = 0.25), indicating potential neuroprotection. Depressive (PHQ‐9: *β* = −0.32) and anxiety (STAI: *β* = −0.08) symptoms significantly declined with higher DI‐GM scores. Sleep quality (PSQI) showed a weak and nonsignificant trend across DI‐GM scores (*β* = 0.04; 95% CI: −0.06 to 0.15; P for trend = 0.40). These findings suggest that adherence to a microbiota‐targeted diet is independently associated with better metabolic, inflammatory, and psychological health outcomes.

**FIGURE 2:**
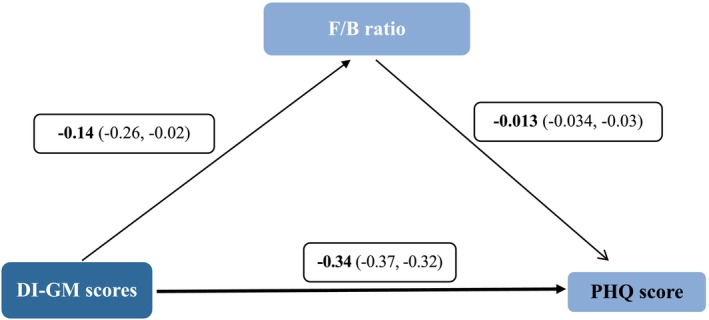
Path analysis model illustrating the relationships between the F/B ratio, Dietary Index for Gut Microbiota (DI‐GM) scores, and PHQ score. Standardized coefficients are presented. The model was adjusted for age, sex, BMI, total energy intake, and physical activity (METs). Solid lines represent statistically significant pathways (*p* < 0.05), and dashed lines indicate nonsignificant pathways.

Figure 2 presents a path analysis model demonstrating the relationships between F/B ratio, DI‐GM scores, and PHQ score. The results indicate a statistically significant negative association between F/B ratio and DI‐GM scores (*β* = −0.14, 95% CI: −0.26, −0.02), suggesting that higher F/B ratios are linked to lower DI‐GM scores. The relationship between F/B ratio and PHQ score was weak and potentially nonsignificant (*β* = −0.013, 95% CI: −0.034, −0.03). In contrast, DI‐GM scores showed a strong and significant negative relationship with PHQ score (*β* = −0.34, 95% CI: −0.37, −0.32), indicating that higher DI‐GM scores are consistently associated with lower PHQ scores. These findings highlight the indirect influence of the F/B ratio on PHQ scores through its effect on DI‐GM scores, with the strongest pathway being the direct effect of DI‐GM scores on PHQ score.

### 3.5 Mediation Role of F/B Ratio in the Relationship Between DI‐GM Score and Health Outcomes

Table 5 summarizes the mediation analysis evaluating whether the F/B ratio mediates the relationship between the DI‐GM score and selected health outcomes. A higher DI‐GM score was directly associated with lower depression (PHQ‐9; *β* = −0.34, 95% CI: −0.37 to −0.32) and anxiety scores (STAI; *β* = −2.51, 95% CI: −2.68 to −2.33), with small but significant indirect effects through the F/B ratio (PHQ‐9: *β* = −0.0013, STAI: *β* = −0.07), indicating partial mediation. For CRP, although the DI‐GM score was strongly and inversely associated (*β* = −0.11, 95% CI: −0.11 to −0.10), the indirect effect via the F/B ratio was not significant. In contrast, for BDNF, a positive direct effect was observed (*β* = 0.63, 95% CI: 0.58 to 0.68), accompanied by a small but significant negative indirect effect (*β* = −0.01), suggesting a minor suppressor role of the F/B ratio. Regarding sleep quality (PSQI), a modest direct positive association with the DI‐GM score was found (*β* = 0.04), while the indirect effect through the F/B ratio was borderline (*β* = −0.026, 95% CI: −0.076 to 0.002). These findings suggest partial mediation by the F/B ratio in mental health outcomes, with limited effects on inflammatory and neurotrophic markers. The mediation models demonstrated acceptable to excellent fit based on established criteria. For mental health outcomes (PHQ‐9 and STAI), the structural models showed good fit with RMSEA = 0.034, CFI = 0.96, TLI = 0.94, and SRMR = 0.038, indicating that the hypothesized mediation via the F/B ratio fit the observed data well. For CRP, BDNF, and sleep quality models, which were just identified, fit indices were not applicable, reflecting a perfect fit by definition. These results support the validity of the specified mediation pathways in the analyzes. Overall, the fit indices support the adequacy and robustness of the hypothesized mediation models and the validity of the reported direct and indirect effects.

**TABLE 5:** Mediation analysis: Role of F/B ratio in the relationship between DI‐GM score and outcomes.

Outcome variables	Path	Effect type	Estimate (95% CI)	*p*	Model fit indices
PHQ‐9	DI‐GM → PHQ	Direct Effect	−0.34 (−0.37, −0.32)	< 0.001	RMSEA = 0.034 CFI = 0.96 TLI = 0.94 SRMR = 0.038
	DI‐GM → F/B ratio → PHQ	Indirect Effect	−0.013 (−0.034, −0.03)	0.01*	
	Total effect	−0.34 (−0.37, −0.32)		< 0.001	
STAI	DI‐GM → STAI	Direct Effect	−2.51 (−2.68, −2.33)	< 0.001	RMSEA = 0.034 CFI = 0.96 TLI = 0.94 SRMR = 0.038
	DI‐GM → F/B ratio → STAI	Indirect Effect	−0.07 (−0.13, −0.02)	0.02*	
	Total effect	−2.57 (−2.73, −2.42)		< 0.001	
CRP	DI‐GM → CRP	Direct Effect	−0.11 (−0.11, −0.10)	< 0.001	Not applicable—Just‐identified model*
	DI‐GM → F/B ratio → CRP	Indirect Effect	< 0.014 (−0.009, 0.017)	0.32	
	Total effect	−0.11 (−0.11, −0.10)		< 0.001	
BDNF	DI‐GM → BDNF	Direct Effect	0.63 (0.58, 0.68)	< 0.001	Not applicable—Just‐identified model*
	DI‐GM → F/B ratio → BDNF	Indirect Effect	−0.01 (−0.02, −0.015)	0.01*	
	Total effect	0.63 (0.58, 0.68)		< 0.001	
Sleep quality (PSQI)	DI‐GM → Sleep Quality	Direct Effect	0.04 (0.01, 0.06)	0.04*	Not applicable—Just‐identified model*
	DI‐GM → F/B ratio → Sleep Quality	Indirect Effect	−0.026 (−0.076, 0.002)	0.07	
	Total effect	0.03 (0.01, 0.06)		0.03*	

*Note:* *β*‐coefficients represent the estimated direct, indirect, and total effects of DI‐GM score on each outcome via F/B ratio. The *indirect effect* was calculated as the product of the coefficient from DI‐GM → F/B ratio and F/B ratio → outcome. All models were fitted using maximum likelihood estimation in *Stata/SE 18.0*. Confidence intervals (95%) that *do not include zero* indicate statistically significant associations at the *p* < 0.05 level. The models were adjusted for *age, sex, BMI, total energy intake*, and *total physical activity (METs)*. *Negative coefficients* indicate a decrease in the outcome with increasing DI‐GM score; *positive coefficients* indicate an increase. *Model fit indices* (RMSEA, CFI, TLI, SRMR) are only reported where the model has degrees of freedom. For *just‐identified models*, these are not applicable as such models are saturated and always show perfect fit by definition.

* Indicates statistical significance at *p* < 0.05.

Collectively, these data demonstrate that a higher DI‐GM score is independently associated with improved metabolic, inflammatory, cardiovascular, and mental health outcomes. These associations are largely preserved after adjustment for key confounders, supporting the DI‐GM score as a meaningful nutritional tool. Furthermore, the F/B ratio partially mediates the beneficial effects of the DI‐GM score on depression and anxiety, highlighting the potential importance of gut microbiota in the gut‐brain axis.

## 4 Discussion

To our knowledge, this is the first study to link the DI‐GM to both inflammatory and tumor biomarkers, as well as neurocognitive indicators like BDNF, in a large adult sample. The present study provides robust evidence supporting the relevance and applicability of the DI‐GM score as a novel, integrative dietary index linked to a broad spectrum of favorable health outcomes. By analyzing dietary intake, biochemical markers, gut microbiota proxies, and mental health indicators in a cross‐sectional cohort of 650 adults, we demonstrate that a higher DI‐GM score—reflecting adherence to a fiber‐rich, nutrient‐dense, and anti‐inflammatory dietary pattern—is consistently associated with improved metabolic, cardiovascular, inflammatory, and neurocognitive profiles. Specifically, participants with higher DI‐GM scores exhibited significantly lower cardiometabolic risk markers and improved insulin sensitivity. Inflammatory biomarkers, including CRP and calprotectin, were also significantly reduced, indicating lower systemic inflammation—a factor potentially contributing to improved mood and reduced risk of depression. Moreover, higher DI‐GM scores were associated with elevated levels of BDNF, suggesting enhanced neurocognitive function. Expanding upon these novel findings, participants with higher DI‐GM scores also showed lower levels of CEA, implying a potential reduction in colorectal cancer risk. Collectively, these results underscore the critical role of dietary patterns and gut microbiota composition in modulating systemic inflammation, psychological well‐being, and tumor‐related biomarkers.

Participants with higher DI‐GM scores consumed diets lower in total energy, saturated fats, and carbohydrates, yet significantly higher in dietary fiber—aligning with the principles of diets known to confer systemic and gut‐specific health benefits, such as the Mediterranean, MIND, and plant‐forward dietary patterns. The inverse relationship between the DI‐GM score and caloric/macronutrient intake, coupled with increased fiber density, suggests that this score captures key anti‐inflammatory and microbiota‐supportive dietary components (Miketinas et al. 2019). Given that fiber is a major substrate for short‐chain fatty acid (SCFA) production, which modulates intestinal barrier integrity and immune regulation, the observed benefits of the DI‐GM score are biologically plausible and mechanistically grounded (Fusco et al. 2023).

### 4.1 Cardiometabolic Health: Glycemic and Lipid Improvements

Metabolic and cardiovascular markers improved significantly with increasing DI‐GM scores. Notably, fasting glucose, insulin, and HOMA‐IR were markedly lower in participants with higher scores, even after controlling for age, sex, BMI, energy, and fiber intake. These findings support the notion that DI‐GM‐associated diets may promote insulin sensitivity and glycemic regulation, potentially through multiple pathways, including reduced postprandial glycemic load, improved adipokine signaling, and decreased systemic inflammation (Papakonstantinou et al. 2022).

Lipid metabolism also exhibited favorable trends, with significant reductions in LDL‐C, total cholesterol, and triglycerides, and an increase in HDL‐C. These outcomes are consistent with the role of dietary fiber in cholesterol binding and excretion, as well as the anti‐atherogenic properties of unsaturated fats, phytochemicals, and polyphenols often found in high‐DI‐GM diets (Islam et al. 2021). Moreover, the reduction in blood pressure across quartiles, although modest, suggests improved endothelial function and vascular tone, possibly mediated by decreased sodium intake or improved nitric oxide bioavailability (Godos et al. 2019).

### 4.2 Inflammation and Gut‐Immune Interactions

Systemic and intestinal inflammatory markers—including CRP, LPS, and TMAO—were significantly lower among those with higher DI‐GM scores, supporting the anti‐inflammatory nature of this dietary pattern (Kase et al. 2024; Zhang et al. 2024). These findings align with previous studies that demonstrated similar reductions in inflammatory markers among individuals adhering to Mediterranean and plant‐based diets, which are also known for their anti‐inflammatory properties (Bolori et al. 2019). These biomarkers are implicated in a range of chronic diseases, including atherosclerosis, diabetes, colorectal cancer, and neurodegeneration (Mostafavi Abdolmaleky and Zhou 2024; Xie et al. 2025). Particularly, CRP showed a strong inverse association with the DI‐GM score, even after multivariable adjustment, suggesting a diet‐related attenuation of low‐grade systemic inflammation. These biomarkers are implicated in a wide array of chronic diseases, ranging from atherosclerosis and type 2 diabetes to colorectal cancer and neurodegenerative disorders (Luan and Yao 2018). CRP, a classical marker of low‐grade systemic inflammation, showed a robust negative correlation with the DI‐GM score, indicating that adherence to this dietary pattern may help mitigate chronic inflammatory burden. This effect may be attributed to reduced translocation of bacterial endotoxins such as LPS, which can trigger innate immune activation and contribute to metabolic endotoxemia (Moludi et al. 2020).

Indeed, LPS levels were significantly lower in higher DI‐GM score groups, suggesting improved gut barrier integrity, potentially via increased SCFA production and mucus stabilization (Fan et al. 2021). TMAO—a gut‐derived metabolite linked to cardiovascular disease—also decreased with higher DI‐GM scores, indicating reduced availability of TMA precursors (e.g., choline) or shifts toward less TMA‐producing microbial communities. These anti‐inflammatory effects align with findings from prebiotic and probiotic studies (Mostafavi Abdolmaleky and Zhou 2024; Moludi et al. 2020), but the DI‐GM score uniquely reflects habitual dietary patterns at the population level. The strong inverse link between DI‐GM scores and TMAO highlights the diet's potential to modulate gut microbial metabolism and reduce cardiovascular risk through both dietary intake and microbiota changes.

Importantly, our findings also show that calprotectin and CEA levels decrease with increasing DI‐GM score, implying reduced gut mucosal inflammation and potential mitigation of colorectal neoplastic risk. Similar results have been reported in studies examining high‐fiber diets, which also showed lower levels of calprotectin and CEA (Fritsch et al. 2021), indicating a protective effect against colorectal cancer. Calprotectin, a calcium‐binding protein released by activated neutrophils, is a well‐established biomarker of intestinal inflammation, often used clinically to differentiate irritable bowel syndrome from inflammatory bowel disease (Konikoff and Denson 2006). Lower calprotectin levels indicate reduced immune cell infiltration into the gut epithelium, which is consistent with improved mucosal immunity under a high‐DI‐GM diet (Nisapakultorn et al. 2001). Similarly, decreased CEA—a tumor‐associated glycoprotein whose elevation is linked to gastrointestinal malignancies (Campos‐da‐Paz et al. 2018)—suggests that DI‐GM‐aligned diets may confer protective effects against colorectal carcinogenesis. These findings align with previous research showing that fiber‐rich diets reduce intestinal permeability and pathobiont expansion, thereby preserving epithelial homeostasis and reducing oncogenic risk (Nakamura et al. 2023).

### 4.3 Gut Microbiota: The Role of the F/B Ratio

An important aspect of this study is the exploration of gut microbiota composition through the F/B ratio, a commonly used but somewhat controversial marker of dysbiosis (Rutsch et al. 2020). Previous studies have shown that lower F/B ratios are often associated with improved metabolic health, particularly in individuals following a high‐fiber diet (Mostafavi Abdolmaleky and Zhou 2024; Moludi et al. 2020; Nakamura et al. 2023). Our findings indicate a statistically significant decrease in the F/B ratio with higher DI‐GM scores, suggesting a shift toward a more balanced microbial ecosystem. While the F/B ratio is a crude proxy, its association with metabolic and inflammatory states in prior studies lends credence to its utility as a population‐level biomarker. However, the observed decrease in alpha diversity with higher DI‐GM scores—measured via the Shannon Index—was unexpected. It may reflect increased dominance of specific beneficial taxa rather than a reduction in overall microbial health (Keirns et al. 2024), underscoring the need for more detailed taxonomic and functional microbiome profiling in future studies.

### 4.4 Mental Health and Neurotrophic Outcomes

The observed improvements in depressive and anxiety symptoms with higher DI‐GM scores highlight the potential psychotropic and neuroprotective roles of diet. These results corroborate findings from studies linking the Mediterranean diet to lower rates of depression and anxiety, suggesting that dietary patterns significantly influence mental health (Mofrad et al. 2019; Bolori et al. 2019). These findings are supported by growing evidence linking dietary patterns to mental health via the gut‐brain axis. Notably, BDNF—a key neurotrophin involved in synaptic plasticity, neurogenesis, and mood regulation (Ninan 2014)—was significantly elevated in participants with higher DI‐GM scores. Given that BDNF expression is modulated by inflammation, oxidative stress, and gut microbiota metabolites (e.g., SCFAs), this association suggests a plausible mechanistic pathway linking diet to improved mental well‐being (Kolodziejczyk et al. 2019; Jian et al. 2021; Zeng et al. 2017).

Although sleep quality, as measured by the PSQI, improved modestly across quartiles, the lack of statistical significance indicates that either the effect size is small or that other unmeasured variables (e.g., caffeine, light exposure, chronotype) may exert stronger influences on sleep regulation than diet alone.

### 4.5 Mediation Analysis: Gut Microbiota as a Mechanistic Link

Mediation analysis revealed that the F/B ratio partially mediated the relationship between DI‐GM score and psychological outcomes, including depression and anxiety. This finding is consistent with other studies that have shown a mediating role of gut microbiota in the relationship between diet and mental health outcomes (Estrada and Contreras 2019; Maranduba et al. 2015). Although the indirect effects were small, they were statistically significant, suggesting that diet‐induced modulation of microbial composition may contribute to improved mental health. Interestingly, the F/B ratio did not significantly mediate the relationship between DI‐GM score and inflammatory or neurotrophic biomarkers, implying that the diet's anti‐inflammatory and neuroprotective effects may operate through microbiota‐independent mechanisms as well. For BDNF, the negative indirect effect observed via the F/B ratio suggests a minor suppressor effect, which may reflect a complex interplay between microbial signaling and host neurobiology. This complexity has been noted in previous studies, where the relationship between gut microbiota and neurotrophic factors was found to be influenced by various dietary components (Kolodziejczyk et al. 2019; Kase et al. 2024).

### 4.6 Strengths and Limitations

This study benefits from a relatively large sample size, extensive biomarker profiling, and the integration of dietary, metabolic, inflammatory, and psychological data. The use of multivariable‐adjusted and mediation models enhances the rigor and interpretability of our findings. However, several limitations warrant consideration. First, the cross‐sectional design limits causal inference, and longitudinal or interventional studies are needed to establish temporal and mechanistic relationships. Second, the reliance on the F/B ratio as a proxy for gut microbiota composition is a simplification that may overlook important taxonomic and functional dynamics. Future studies incorporating metagenomic or metabolomic analyzes would provide more detailed insights. Third, residual confounding cannot be ruled out, despite adjustment for key sociodemographic and lifestyle variables.

## 5 Conclusions

In summary, the DI‐GM score is a comprehensive dietary index associated with improved cardiometabolic, inflammatory, gut microbiota, and neurocognitive outcomes. These findings highlight the role of diet in modulating the gut–immune–brain axis and suggest potential benefits for both chronic disease prevention and mental health. Remarkably, reduced inflammatory markers linked to depression and increased BDNF levels point to a protective effect on mood and cognition. Notably, the reductions in calprotectin and CEA levels suggest a potential protective effect against colorectal cancer—representing a novel and clinically relevant contribution of this work. Partial mediation by gut microbiota underscores the importance of microbial mechanisms. These results support the integration of gut‐targeted dietary strategies into public health and personalized nutrition, with future studies needed to evaluate long‐term impacts and gene–environment interactions.

## Author Contributions

Yaqin Meng and Weiyu Ma contributed equally to this work. Yaqin Meng was responsible for the conceptualization, data collection, and manuscript drafting. Weiyu Ma contributed to methodology, data analysis, and manuscript revision. Xiuxiu Li participated in supervision, critical review, and editing of the manuscript. Ninggang Zhang provided overall guidance, supervision, and final approval of the version to be published. All authors read and approved the final manuscript.

## Disclosure

Declaration of Generative AI and AI‐assisted technologies in the writing process: Nothing to disclose. No generative artificial intelligence (AI) or AI‐assisted technologies were used in the writing of this manuscript.

## Ethics Statement

This study was approved by the Medical Research Ethics Committee of Shanxi Province Cancer Hospital (Approval No. 2024‐417). Written informed consent was obtained from all participants. All procedures adhered to the principles of the Declaration of Helsinki.

## Consent

All authors approved the final manuscript and consented to its submission to the journal.

## Conflicts of Interest

The authors declare no conflicts of interest.

## Data Availability

All data generated or analyzed during this study are included in the manuscript. Additional data are available upon reasonable request from the corresponding author.

## References

[fsn370951-bib-0001] Ağagündüz, D. , E. Cocozza , Ö. Cemali , et al. 2023. “Understanding the Role of the Gut Microbiome in Gastrointestinal Cancer: A Review.” Frontiers in Pharmacology 14: 1130562. 36762108 10.3389/fphar.2023.1130562 PMC9903080

[fsn370951-bib-0002] Annunziata, G. , A. Arnone , R. Ciampaglia , G. C. Tenore , and E. Novellino . 2020. “Fermentation of Foods and Beverages as a Tool for Increasing Availability of Bioactive Compounds. *Focus on Short‐Chain Fatty Acids* .” Food 9, no. 8: 999. 10.3390/foods9080999 PMC7466228 32722417

[fsn370951-bib-0003] Bengmark, S. 2013. “Processed Foods, Dysbiosis, Systemic Inflammation, and Poor Health.” Current Nutrition & Food Science 9, no. 2: 113–143.

[fsn370951-bib-0004] Bianchetti, G. , F. de Maio , A. Abeltino , et al. 2023. “Unraveling the Gut Microbiome–Diet Connection: Exploring the Impact of Digital Precision and Personalized Nutrition on Microbiota Composition and Host Physiology.” Nutrients 15, no. 18: 3931. 37764715 10.3390/nu15183931 PMC10537332

[fsn370951-bib-0005] Bolori, P. , L. Setaysh , N. Rasaei , F. Jarrahi , and M. Saeid Yekaninejad . 2019. “Adherence to a [Mofrad, 2019 #21;Bolori, 2019 #41]Healthy Plant Diet May Reduce Inflammatory Factors in Obese and Overweight Women—A Cross‐Sectional Study.” Diabetes and Metabolic Syndrome: Clinical Research and Reviews 13, no. 4: 2795–2802. 10.1016/j.dsx.2019.07.019 31405709

[fsn370951-bib-0006] Buttó, L. F. , and D. Haller . 2016. “Dysbiosis in Intestinal Inflammation: Cause or Consequence.” International Journal of Medical Microbiology 306, no. 5: 302–309. 27012594 10.1016/j.ijmm.2016.02.010

[fsn370951-bib-0007] Campos‐da‐Paz, M. , J. G. Dórea , A. S. Galdino , Z. G. M. Lacava , and M. de Fatima Menezes Almeida Santos . 2018. “Carcinoembryonic Antigen (CEA) and Hepatic Metastasis in Colorectal Cancer: Update on Biomarker for Clinical and Biotechnological Approaches.” Recent Patents on Biotechnology 12, no. 4: 269–279. 30062978 10.2174/1872208312666180731104244

[fsn370951-bib-0008] Cheng, Y. , Z. Zhu , Z. Yang , et al. 2025. “Alterations in Fecal Microbiota Composition and Cytokine Expression Profiles in Adolescents With Depression: A Case‐Control Study.” Scientific Reports 15, no. 1: 12177. 40204825 10.1038/s41598-025-97369-6 PMC11982373

[fsn370951-bib-0009] Costa, M. , and J. S. Weese . 2019. “Methods and Basic Concepts for Microbiota Assessment.” Veterinary Journal 249: 10–15. 31239159 10.1016/j.tvjl.2019.05.005

[fsn370951-bib-0010] Dadfar, M. , N. Momeni Safarabad , A. A. Asgharnejad Farid , M. Nemati Shirzy , and F. Ghazie pour Abarghouie . 2018. “Reliability, Validity, and Factorial Structure of the World Health Organization‐5 Well‐Being Index (WHO‐5) in Iranian Psychiatric Outpatients.” Trends in Psychiatry and Psychotherapy 40: 79–84. 29995154 10.1590/2237-6089-2017-0044

[fsn370951-bib-0011] Du, Q. , H. Liu , C. Yang , X. Chen , and X. Zhang . 2022. “The Development of a Short Chinese Version of the State‐Trait Anxiety Inventory.” Frontiers in Psychiatry 13: 854547. 35619610 10.3389/fpsyt.2022.854547 PMC9128482

[fsn370951-bib-0012] Effatpanah, M. , A. Nakhostin‐Ansari , F. Gorgani , et al. 2024. “Burden and Epidemiology of Mental Disorders in the Middle East and North Africa From 1990 to 2019: Findings From the Global Burden of Disease Study.” Balkan Medical Journal 41, no. 2: 121–129. 38332586 10.4274/balkanmedj.galenos.2024.2023-11-55 PMC10913114

[fsn370951-bib-0013] Estrada, J. A. , and I. Contreras . 2019. “Nutritional Modulation of Immune and Central Nervous System Homeostasis: The Role of Diet in Development of Neuroinflammation and Neurological Disease.” Nutrients 11, no. 5: 1076. 31096592 10.3390/nu11051076 PMC6566411

[fsn370951-bib-0014] Fan, S. , Z. Zhang , Y. Zhong , et al. 2021. “Microbiota‐Related Effects of Prebiotic Fibres in Lipopolysaccharide‐Induced Endotoxemic Mice: Short Chain Fatty Acid Production and Gut Commensal Translocation.” Food & Function 12, no. 16: 7343–7357. 34180493 10.1039/d1fo00410g

[fsn370951-bib-0015] Fritsch, J. , L. Garces , M. A. Quintero , et al. 2021. “Low‐Fat, High‐Fiber Diet Reduces Markers of Inflammation and Dysbiosis and Improves Quality of Life in Patients With Ulcerative Colitis.” Clinical Gastroenterology and Hepatology 19, no. 6: 1189–1199.e30. 32445952 10.1016/j.cgh.2020.05.026

[fsn370951-bib-0016] Fusco, W. , M. B. Lorenzo , M. Cintoni , et al. 2023. “Short‐Chain Fatty‐Acid‐Producing Bacteria: Key Components of the Human Gut Microbiota.” Nutrients 15, no. 9: 2211. 37432351 10.3390/nu15092211 PMC10180739

[fsn370951-bib-0017] Godos, J. , M. Vitale , A. Micek , et al. 2019. “Dietary Polyphenol Intake, Blood Pressure, and Hypertension: A Systematic Review and Meta‐Analysis of Observational Studies.” Antioxidants 8, no. 6: 152. 31159186 10.3390/antiox8060152 PMC6616647

[fsn370951-bib-0018] Islam, S. U. , M. B. Ahmed , H. Ahsan , and Y. S. Lee . 2021. “Recent Molecular Mechanisms and Beneficial Effects of Phytochemicals and Plant‐Based Whole Foods in Reducing LDL‐C and Preventing Cardiovascular Disease.” Antioxidants 10, no. 5: 784. 34063371 10.3390/antiox10050784 PMC8157003

[fsn370951-bib-0019] Jian, C. , P. Luukkonen , S. Sädevirta , H. Yki‐Järvinen , and A. Salonen . 2021. “Impact of Short‐Term Overfeeding of Saturated or Unsaturated Fat or Sugars on the Gut Microbiota in Relation to Liver Fat in Obese and Overweight Adults.” Clinical Nutrition 40, no. 1: 207–216. 32536582 10.1016/j.clnu.2020.05.008

[fsn370951-bib-0020] Kase, B. E. , A. D. Liese , J. Zhang , E. A. Murphy , L. Zhao , and S. E. Steck . 2024. “The Development and Evaluation of a Literature‐Based Dietary Index for Gut Microbiota.” Nutrients 16, no. 7: 1045. 38613077 10.3390/nu16071045 PMC11013161

[fsn370951-bib-0021] Keirns, B. H. , A. R. Medlin , K. A. Maki , et al. 2024. “Biomarkers of Intestinal Permeability Are Associated With Inflammation in Metabolically Healthy Obesity but Not Normal‐Weight Obesity.” American Journal of Physiology. Heart and Circulatory Physiology 327, no. 5: H1135–H1145. 39212768 10.1152/ajpheart.00381.2024 PMC11901334

[fsn370951-bib-0022] Kolodziejczyk, A. A. , D. Zheng , and E. Elinav . 2019. “Diet–Microbiota Interactions and Personalized Nutrition.” Nature Reviews Microbiology 17, no. 12: 742–753. 31541197 10.1038/s41579-019-0256-8

[fsn370951-bib-0023] Konikoff, M. R. , and L. A. Denson . 2006. “Role of Fecal Calprotectin as a Biomarker of Intestinal Inflammation in Inflammatory Bowel Disease.” Inflammatory Bowel Diseases 12, no. 6: 524–534. 16775498 10.1097/00054725-200606000-00013

[fsn370951-bib-0024] Luan, Y.‐y. , and Y.‐m. Yao . 2018. “The Clinical Significance and Potential Role of C‐Reactive Protein in Chronic Inflammatory and Neurodegenerative Diseases.” Frontiers in Immunology 9: 1302. 29951057 10.3389/fimmu.2018.01302 PMC6008573

[fsn370951-bib-0025] Manea, L. , S. Gilbody , and D. McMillan . 2015. “A Diagnostic Meta‐Analysis of the Patient Health Questionnaire‐9 (PHQ‐9) Algorithm Scoring Method as a Screen for Depression.” General Hospital Psychiatry 37, no. 1: 67–75. 25439733 10.1016/j.genhosppsych.2014.09.009

[fsn370951-bib-0026] Maranduba, C. M. D. C. , S. B. R. De Castro , G. T. D. Souza , et al. 2015. “Intestinal Microbiota as Modulators of the Immune System and Neuroimmune System: Impact on the Host Health and Homeostasis.” Journal of Immunology Research 2015, no. 1: 931574. 25759850 10.1155/2015/931574 PMC4352473

[fsn370951-bib-0027] Miketinas, D. C. , G. A. Bray , R. A. Beyl , D. H. Ryan , F. M. Sacks , and C. M. Champagne . 2019. “Fiber Intake Predicts Weight Loss and Dietary Adherence in Adults Consuming Calorie‐Restricted Diets: The POUNDS Lost (Preventing Overweight Using Novel Dietary Strategies) Study.” Journal of Nutrition 149, no. 10: 1742–1748. 31174214 10.1093/jn/nxz117 PMC6768815

[fsn370951-bib-0028] Mofrad, M. D. , F. Siassi , B. Guilani , N. Bellissimo , and L. Azadbakht . 2019. “Association of Dietary Phytochemical Index and Mental Health in Women: A Cross‐Sectional Study.” British Journal of Nutrition 121, no. 9: 1049–1056. 30714542 10.1017/S0007114519000229

[fsn370951-bib-0029] Mollayeva, T. , P. Thurairajah , K. Burton , S. Mollayeva , C. M. Shapiro , and A. Colantonio . 2016. “The Pittsburgh Sleep Quality Index as a Screening Tool for Sleep Dysfunction in Clinical and Non‐Clinical Samples: A Systematic Review and Meta‐Analysis.” Sleep Medicine Reviews 25: 52–73. 26163057 10.1016/j.smrv.2015.01.009

[fsn370951-bib-0030] Moludi, J. , V. Maleki , H. Jafari‐Vayghyan , E. Vaghef‐Mehrabany , and M. Alizadeh . 2020. “Metabolic Endotoxemia and Cardiovascular Disease: A Systematic Review About Potential Roles of Prebiotics and Probiotics.” Clinical and Experimental Pharmacology and Physiology 47, no. 6: 927–939. 31894861 10.1111/1440-1681.13250

[fsn370951-bib-0031] Mostafavi Abdolmaleky, H. , and J.‐R. Zhou . 2024. “Gut Microbiota Dysbiosis, Oxidative Stress, Inflammation, and Epigenetic Alterations in Metabolic Diseases.” Antioxidants 13, no. 8: 985. 39199231 10.3390/antiox13080985 PMC11351922

[fsn370951-bib-0032] Nakamura, Y. K. , C. Metea , V. Llorenç , L. Karstens , A. Balter , and P. Lin . 2023. “A Diet Rich in Fermentable Fiber Promotes Robust Changes in the Intestinal Microbiota, Mitigates Intestinal Permeability, and Attenuates Autoimmune Uveitis.” Scientific Reports 13, no. 1: 10806. 37402809 10.1038/s41598-023-37062-8 PMC10319740

[fsn370951-bib-0033] Ninan, I. 2014. “Synaptic Regulation of Affective Behaviors; Role of BDNF.” Neuropharmacology 76: 684–695. 23747574 10.1016/j.neuropharm.2013.04.011 PMC3825795

[fsn370951-bib-0034] Nisapakultorn, K. , K. F. Ross , and M. C. Herzberg . 2001. “Calprotectin Expression Inhibits Bacterial Binding to Mucosal Epithelial Cells.” Infection and Immunity 69, no. 6: 3692–3696. 11349032 10.1128/IAI.69.6.3692-3696.2001 PMC98370

[fsn370951-bib-0035] Papakonstantinou, E. , C. Oikonomou , G. Nychas , and G. D. Dimitriadis . 2022. “Effects of Diet, Lifestyle, Chrononutrition and Alternative Dietary Interventions on Postprandial Glycemia and Insulin Resistance.” Nutrients 14, no. 4: 823. 35215472 10.3390/nu14040823 PMC8878449

[fsn370951-bib-0036] Rutsch, A. , J. B. Kantsjö , and F. Ronchi . 2020. “The Gut‐Brain Axis: How Microbiota and Host Inflammasome Influence Brain Physiology and Pathology.” Frontiers in Immunology 11: 604179. 33362788 10.3389/fimmu.2020.604179 PMC7758428

[fsn370951-bib-0037] Spielberger, C. D. , and R. L. Rickman . 1988. Assessment of State and Trait Anxiety. Taylor & Francis.

[fsn370951-bib-0038] Wang, L. , Y. X. Wu , Y. Q. Lin , et al. 2022. “Reliability and Validity of the Pittsburgh Sleep Quality Index Among Frontline COVID‐19 Health Care Workers Using Classical Test Theory and Item Response Theory.” Journal of Clinical Sleep Medicine 18, no. 2: 541–551. 34534069 10.5664/jcsm.9658 PMC8805004

[fsn370951-bib-0039] Xie, H. , J. Jiang , S. Cao , et al. 2025. “The Role of Gut Microbiota‐Derived Trimethylamine N‐Oxide in the Pathogenesis and Treatment of Mild Cognitive Impairment.” International Journal of Molecular Sciences 26, no. 3: 1373. 39941141 10.3390/ijms26031373 PMC11818489

[fsn370951-bib-0040] Zeng, M. , N. Inohara , and G. Nuñez . 2017. “Mechanisms of Inflammation‐Driven Bacterial Dysbiosis in the Gut.” Mucosal Immunology 10, no. 1: 18–26. 27554295 10.1038/mi.2016.75 PMC5788567

[fsn370951-bib-0041] Zhang, X. , Q. Yang , J. Huang , H. Lin , N. Luo , and H. Tang . 2024. “Association of the Newly Proposed Dietary Index for Gut Microbiota and Depression: The Mediation Effect of Phenotypic Age and Body Mass Index.” European Archives of Psychiatry and Clinical Neuroscience 275: 1–12. 10.1007/s00406-024-01912-x 39375215

[fsn370951-bib-0042] Zhou, Y. , Y. Zhang , S. Jin , J. Lv , M. Li , and N. Feng . 2024. “The Gut Microbiota Derived Metabolite Trimethylamine N‐Oxide: Its Important Role in Cancer and Other Diseases.” Biomedicine & Pharmacotherapy 177: 117031. 38925016 10.1016/j.biopha.2024.117031

